# Impact of ADMA, endothelial progenitor cells and traditional cardiovascular risk factors on pulse wave velocity among prediabetic individuals

**DOI:** 10.1186/1475-2840-11-141

**Published:** 2012-11-15

**Authors:** Ioannis Protopsaltis, Stefanos Foussas, Angeliki Angelidi, Angelos Gritzapis, Theodoros Ν Sergentanis, Spyros Matsagos, Konstantinos Tzirogiannis, Georgios I Panoutsopoulos, Georgios Dimitriadis, Sotirios Raptis, Andreas Melidonis

**Affiliations:** 1 Diabetes Center, Tzanio General Hospital of Piraeus, Zanni and Afendouli 1, Piraeus, 18537, Greece; 2 Department of Cardiology, Tzanio General Hospital of Piraeus, Piraeus, Greece; 3 Laboratory of Cellular Biology and Immunology, Locus Medicus S.A, Athens, Greece; 4 Department of Hygiene, Epidemiology and Medical Statistics, Medical School, National University of Athens, Athens, Greece; 5 Blood Bank Service, Tzanio General Hospital of Piraeus, Piraeus, Greece; 6 Department of Nursing, University of Peloponnese, Sparta, Greece; 7 2nd Department of Internal Medicine, Research Institute and Diabetes Center, ‘Attikon’ University General Hospital, Athens, Greece; 8 Hellenic National Diabetic Centre, Athens, Greece

**Keywords:** Pre-diabetes, ADMA, Pulse wave velocity, Endothelial progenitor cells

## Abstract

**Background:**

Central arterial stiffness represents a well-established predictor of cardiovascular disease. Decreased circulating endothelial progenitor cells (EPCs), increased asymmetric dimethyl-arginine (ADMA) levels, traditional cardiovascular risk factors and insulin resistance have all been associated with increased arterial stiffness. The correlations of novel and traditional cardiovascular risk factors with central arterial stiffness in prediabetic individuals were investigated in the present study.

**Methods:**

The study population consisted of 53 prediabetic individuals. Individuals were divided into groups of isolated impaired fasting glucose (IFG), isolated impaired glucose tolerance (IGT) and combined IGT-IFG. Age, sex, family history of diabetes, smoking history, body mass index (BMI), waist to hip ratio (WHR), waist circumference (WC), blood pressure, lipid profile, levels of high sensitive C-reactive protein (hsCRP), glomerular filtration rate (GFR), and history of antihypertensive or statin therapy were obtained from all participants. Insulin resistance was evaluated using the Homeostatic Model Assessment (HOMA-IR). Carotid -femoral pulse wave velocity was used as an index of arterial stiffness. Circulating EPC count and ADMA serum levels were also determined.

**Results:**

Among studied individuals 30 (56.6%) subjects were diagnosed with isolated IFG, 9 (17%) with isolated IGT (17%) and 14 with combined IFG-IGT (26.4%). In univariate analysis age, mean blood pressure, fasting glucose, total cholesterol, LDL cholesterol, and ADMA levels positively correlated with pulse-wave velocity while exercise and GFR correlated negatively. EPC count did not correlate with PWV. In multivariate stepwise regression analysis PWV correlated independently and positively with LDL-Cholesterol (low density lipoprotein) and ADMA levels and negatively with exercise.

**Conclusions:**

Elevated ADMA and LDL-C levels are strongly associated with increased arterial stiffness among pre-diabetic subjects. In contrast exercise inversely correlated with arterial stiffness.

## Introduction

Pre-diabetes confers an increased risk for developing type ΙΙ diabetes and cardiovascular disease [1]. Both isolated impaired fasting glucose (IFG) and impaired glucose tolerance (IGT) are characterized by insulin resistance and impaired insulin secretion [2] and insulin resistance constitutes a major cardiovascular risk factor in diabetic and obese patients. A common pathway, by which insulin resistance leads to increased cardiovascular risk, is increased arterial stiffness, which is directly linked to the development of isolated systolic hypertension, increase in cardiac afterload and finally impairment of coronary perfusion [3]. As a consequence of the above, central arterial stiffness is a well-established independent predictor of cardiovascular events [4]. Large-scale epidemiological studies have well established the independent correlation of insulin resistance with arterial stiffness [5], whereas the latter appears to increase consistently with deterioration of glucose tolerance status [6]. Therefore, impaired fasting glucose (IFG), impaired glucose tolerance (IGT), and frank diabetes are all states of increased arterial stiffness [6].

At the molecular level, insulin resistance is characterized by impairment of Phosphoinositide 3-kinase (PI 3-kinase) signaling pathway, whereas the Mitogen-Activated Protein Kinase (MAPK) pathway is unaffected. Decreased PI 3-kinase signaling leads to attenuated induction of endothelial nitric oxide synthase (eNOs) and decreased NO (nitric oxide) production. Additionally, MAPK overdrive results in increased smooth muscle cell proliferation and concurrent induction of numerous growth factors and other inflammatory mediators, all of which contribute to endothelial cell dysfunction and arterial stiffness aggravation [7]. Insulin resistance has also been reported to up-regulate expression of vascular angiotensin II type 1 receptor which alters extracellular matrix homeostasis leading to accumulation of extracellular matrix proteins which further propagates arterial stiffness [8]. Finally chronic hyperglycemia leads to increased formation of advanced glycation end products (AGEs) which accumulate in the arterial wall, covalently modify and link extracellular proteins leading to fibrosis and increased arterial stiffness [9,10].

Although arterial stiffness has been mainly attributed to alterations of the tunica media (elastin/collagen ratio), intima can also influence arterial elasticity through endothelial cell dysfunction, with consequent reduced NO bioavailability and increased vascular smooth muscle tone [11]. In the above context, restoration of endothelial monolayer by endothelial progenitor cells (EPCs) is crucial for maintaining unimpeded endothelial integrity [12]. Recruitment of EPC from bone marrow is NO mediated and seems to be attenuated in pre-diabetic patients [13] and reduced numbers of EPCs have been associated with impaired arterial elasticity among diabetic patients [13,14]. PI 3-kinase pathway is pivotal in the regulation of EPC proliferation, recruitment and mobilization and its impairment in insulin resistance states seems to be a major molecular pathway implicated in reduced EPC counts and increased arterial stiffness [15].

Asymmetric dimethyl-l-arginine (ADMA), an endogenous nitric oxide synthase inhibitor has been associated with endothelial cell dysfunction, decreased EPC proliferation, mobilization and recruitment [16,17] and vascular stiffening [18]. ADMA levels are increased in individuals with insulin resistance and diabetes and hyperglycemia directly increases ADMA levels through decreased activity of dimethylarginine dimethylaminohydrolase (DDAH) [19-21].

As the prevalence of pre-diabetes rises with age, there is also an age-related increase in arterial stiffness. Early identification of novel and traditional risk factors associated with increased stiffness is crucial since their control will directly lead to improvement of arterial elasticity. Although a great number of studies has shed light on the correlation between arterial stiffness and established traditional cardiovascular risk factors, there is a lack of studies investigating the effect of novel cardiovascular risk factors, such as ADMA and EPCs, on arterial stiffness, especially in the pre-diabetic state.

In the present study we investigated the relationship between cardiovascular risk factors, novel and traditional, and arterial stiffness as measured by means of PWV, with emphasis placed on novel risk factors, as ADMA and EPCs, among subjects with pre-diabetes.

## Materials and methods

This cross sectional clinical study was conducted in a tertiary urban Greek hospital. The study population consisted of general population subjects recruited in the outpatient diabetes clinic and internal medicine departments of Tzanio General Hospital, Piraeus Greece. The study was approved by the Institution Ethics committee of the hospital and informed consent was obtained on all cases. All participants underwent a75gr oral glucose tolerance test (OGTT) to determine glucose tolerance status according to the criteria of the American Diabetes Association [22]. IFG diagnosis was based on fasting plasma glucose (FPG) value ≥ 100 mg/dL and < 126 mg/dL. Diagnosis of IGT was based on serum glucose concentration 2-h PG ≥ 140 mg/dL and < 200 mg/dL.

Initially, 80 newly diagnosed subjects with pre-diabetes were enrolled. Three of them did not consent to participation in the study and 24 were not included due to the exclusion criteria adopted (see below) leaving finally 53 pre-diabetic subjects that were finally enrolled. Selected individuals were subsequently divided into groups of isolated IFG, isolated IGT and combined IGT-IFG. For all participants the following parameters were determined at baseline: age, sex, family history for diabetes, smoking history, Body Mass Index (BMI), waist to hip ratio (WHR), waist circumference, waist-to-height ratio, (WHtR) blood pressure (systolic and diastolic), total cholesterol, low density lipoprotein (LDL)-cholesterol, high density lipoprotein (HDL)-cholesterol, and glomerular filtration rate (GFR) using the Cockroft-Gault formula.

Insulin resistance was evaluated using the homeostasis model assessment for insulin resistance (HOMA-IR). C-reactive protein (hs-CRP) was measured with high-sensitivity methods using nephelometry (BN II Nephelometer-Siemens).

Exclusion criteria for pre-diabetic subjects included: coronary artery disease, peripheral arterial disease, history of stroke, history of infection in the last month, autoimmune disease, hyper- or hypothyroidism, excess alcohol intake (> 14 drinks/wk for women and > 20 drinks/wk for men), creatinine clearance less than 60 mL/min, secondary hypertension, chronic heart failure (NYHA class III and IV), history of malignancy, hormone replacement therapy and intake of oral contraceptives. No participant was treated with metformin within three months prior to study entry.

Cardiovascular disease was excluded by medical history questionnaire, physical examination and confirmed by electrocardiography, treadmill testing, and thallium 201 cardiac scintigraphy when applicable. Individuals receiving antihypertensive treatment were considered hypertensive regardless of blood pressure measurements. Use of lipid-lowering, medication was also recorded. Participants were classified as exercisers if they reported moderate physical activity (walking at least 150 minutes/week) or otherwise sedentary. Vigorous physical activity was not reported by any participant.

### Evaluation of ADMA concentration

Venous blood samples were collected from participants after overnight fast, by venipuncture of the antecubital vein, into serum tubes. Samples were centrifuged at 10.000 x g for 10 min and the supernatant serum was collected and stored at -20°C. The quantitative determination of ADMA was performed using ADMA Elisa kit (Immundiagnostik A.G, Stubenwald-Allee, Bensheim, Germany).

### Quantification of circulating Endothelial progenitor cells

Venous blood samples were collected after overnight fast and abstention from smoking. EPCs were detected with flow cytometry (surface expression of CD34 and KDR). The antibody cocktail consisted of CD34+FITC (Beckman Coulter, Fullerton, CA), and anti VEGFR (R&D Systems). As the CD34 FITC antibody concentration was not specified in the respective leaflet, both were mixed and titrated in umbilical cord blood in order to define the dilutions that minimized non-specific staining. Following these preliminary experiments, 5ul of CD34 FITC and 3ul of anti VEGFRPE were mixed and added to 50 ul of whole blood. The fixed amount of blood used in combination with BD Trucount tubesT (BD Biosciences, San Jose, CA, USA) containing known numbers of microbeads, allowed us to measure the absolute number of endothelial cells, as described in the literature [23]. The blood specimen was added to the antibody mix by reverse pipetting to improve the accuracy and reproducibility of measurements. Following incubation for 15 min at room temperature in the dark, 450 ul of NH4Cl were added and incubated for 15min at room temperature in the dark. Each specimen was stained in duplicate. 8000 beads of each sample acquired on FacsCan (BD Biosciences) and control samples were analyzed using BD Cell-Quest T software. Control samples stained with isotype-matched antibodies were used to exclude unspecific staining. The gating for CD34+ cells was based on the basic ISHAGE protocol. The gated CD34+ were examined for the expression of VEGFR antigen and the absolute number of EPC was calculated according to the formula: number of events in region containing cell population/number of events in absolute count bead region X number of beads per test/test volume.

### Measurement of pulse wave velocity (PWV)

PWV was assessed by the Bramwell-Hill formula. Carotid-femoral PWV was measured in all patients in supine position after 5 minutes of rest using Complior® waveform analyzer (Colson, Les Lilas, France) [24]. Patients were advised to abstain from smoking or eating for at least 3 hours and for at least 10 hours from alcohol consumption before measurements, according to current recommendations for standardization of subject condition [25]. Pressure waveforms of the Carotid and femoral arteries were recorded using tonometry sensors at the left carotid and femoral arteries. The waveform analyzer reports PWV of the carotid- femoral arterial segments by simultaneously measuring time intervals for pulse wave along the corresponding arterial path. The path length was estimated using a measuring tape. Three consecutive readings were done for each patient, and the average was taken for analysis. All measurements were performed by the same examiner.

### Statistical analysis

For the evaluation of the factors associated with PWV, a standard two-step approach was followed: univariate and multivariate analysis. At the univariate analysis, parametric tests were appropriately implemented, given that PWV followed the normal distribution (as attested by the Shapiro-Wilk test, p=0.308). The factors whose associations with PWV were examined comprised the following: gender, exercise, smoking (yes vs. no and pack-years), subclassification of pre-diabetes (IFG, IGT, IFG+IGT), intake of statins, intake of renin-angiotensin-aldosterone (RAA) axis inhibitors, intake of b-blockers, family history of diabetes mellitus, EPC count, ADMA levels, age, BMI, waist circumference, hip circumference, WHR, WHtR, mean blood pressure (MBP), heart rate (HR), fasting plasma glucose levels, HbA1c levels, insulin levels, HOMA-IR, hs-CRP, total cholesterol, HDL-C, LDL-C, triglyceride levels and GFR.

At the multivariate analysis, stepwise linear regression was performed. As appropriate, factors proven significant at the univariate analysis were tested in the stepwise multivariate model as independent variables; a subset of them (p<0.05) were appropriately retained during the stepwise selection of variables. In case of collinear and conceptually intertwined variables (such as total cholesterol and LDL), alternative models were constructed. Normality of the studentized (jackknifed) residuals was verified using the Shapiro-Wilk test for each model. Statistical analysis was performed using STATA 11.1 statistical software (Stata Corporation, College Station, TX, USA).

## Results

A total of 53 patients were included in this analysis of whom 30 subjects (56.6%) were diagnosed with isolated IFG, 9 subjects (17%) with isolated IGT (17%) and 14 with combined IFG-IGT (26.4%). Patients’ baseline characteristics are presented in Table 1. Regarding gender, 54.7% of the subjects were women and 35.8% smokers (n=19). Mean age was 50.6±11.2 (range: 31–67) years. 18 (34%) of patients were on therapy with RAS blockade and 12 (22.6%) on statin therapy. In relation to exercise status, 23 subjects (43.4%) reported physical exercise, whereas 30 subjects (56.6%) did not.

**Table 1:** Description of the study sample

**Categorical and ordinal Variables**	**n (%)**
Gender	
*Male*	24 (45.3)
*Female*	29 (54.7)
Exercise	
*Yes*	23 (43.4)
*No*	30 (56.6)
Smoking	
*Yes*	19 (35.8)
*No*	34 (64.2)
Subclassification	
*IFG*	30 (56.6)
*IGT*	9 (17.0)
*IFG+IGT*	14 (26.4)
Statins	
*Yes*	12 (22.6)
*No*	41 (77.4)
RAA	
*Yes*	18 (34.0)
*No*	35 (66.0)
b-blockers	
*Yes*	10 (18.9)
*No*	43 (81.1)
Family history of DM	
*Yes*	21 (42.0)
*No*	29 (58.0)
**Continuous Variables**	**Mean ± SD**
EPCs-3 surface markers (cells/mL)	199 ±180
EPCs-2 surface markers (cells/mL)	262 ±210
ADMA (μmol/L)	0.76 ±0.16
Velocity (m/s)	9.1 ±1.4
Age (years)	50.6 ±11.2
BMI (kg/m^2^)	33.6 ±7.0
Waist circumference (cm)	110.4 ±18.0
Hip circumference (cm)	118.9 ±14.0
WHR	0.93 ±0.08
WHtR	0.65 ±0.10
Smoking (packyears)	20.1 ±39.8
SBP (mmHg)	140.6 ±16.1
DBP (mmHg)	85.4 ±10.2
MBP (mmHg)	103.8 ±11.3
HR (bpm)	77 ±11
Fasting glucose	107.5±10.1
HbA1c (%)	5.99±0.22

Univariate correlation analyses were performed to determine which variables were most closely associated with arterial stiffness. In univariate analysis (Table 2) exercise (p=0.009, Student’s *t*-test), age (r= +0.522, p=0.0001), mean blood pressure (MBP) (r=+0.428, p=0.002), fasting plasma glucose (r= +0.449, p=0.001), total cholesterol (r=+0.601, p<0.0001), LDL cholesterol (r=+0.672, p<0.0001), GFR (r= −0.491, p=0.0003), and ADMA (r=+0.508, p=0.0002) were significantly associated with PWV; the correlation with PWV was positive for all aforementioned variables with the exception of GFR and exercise. In contrast gender, CD34+ KDR+ cell counts, HbA1c levels, HOMA index, hs-CRP, anthropometric variables, pre-diabetic status (IFG, IGT and combined IFG and IGT) and the use of statins or any form of antihypertensive treatment (p=0.186 for RAA inhibitors and p=0.347 for beta –blockers) did not significantly correlate with PWV.

**Table 2:** Results of the univariate analysis

**Categorical Variables**	**PWV (mean±SD)**	**p-value**
Gender		0.439^T^
*Male*	8.9±1.5	
*Female*	9.2±1.2	
Exercise		0.009^T^
*Yes*	8.5±1.3	
*No*	9.6±1.4	
Pre-diabetes subcategory		0.215^A^
*IFG*	8.8±1.1	
*IGT*	9.5±2.2	
*IFG+IGT*	9.4±1.4	
Use of statins		0.656^T^
*Yes*	9.3±1.3	
*No*	9.0±1.5	
**Continuous Variables**	**PWV (mean±SD)**	**r§ (p-value)**
Age (years)		+0.522 (0.0001)
<48	8.5±0.9	
≥48	9.6±1.6	
MBP (mmHg)		+0.428 (0.002)
<106.3	8.1±0.8	
≥106.3	10.0±1.3	
Total cholesterol (mg/dL)		+0.601 (<0.0001)
<196	8.2±1.1	
≥196	9.9±1.2	
LDL (mg/dL)		+0.672 (<0.0001)
<118.4	8.0±0.7	
≥118.4	10.1±1.2	
GFR (mL/min)		−0.491 (0.0003)
<77	9.6±1.3	
≥77	8.5±1.3	
ADMA (μmol/L)		+0.508 (0.0002)
<0.757	8.1±0.8	
≥0.757	9.8±1.3	
BMI (kg/m^2^)		−0.164 (0.254)
<32.9	9.2±1.3	
≥32.9	9.0±1.6	
WHR		+0.154 (0.284)
<0.91	8.8±1.2	
≥0.91	9.4±1.6	
CD34+KDR+ (cells/mL)		−0.121 (0.403)
<292	9.3±1.5	
≥292	8.9±1.4	
HOMA-IR		+0.218 (0.128)
<4.51	9.0±1.4	
≥4.51	9.2±1.5	
Fasting glucose (mg/dL)		+0.449 (0.001)
<107	8.6±1.7	
≥107	9.5±1.0	
HbA1c (%)		+0.006 (0.967)
<6	9.0±1.2	
≥6	9.2±1.6	
hs-CRP (mg/L)		+0.018 (0.907)
<3.89	8.9±1.5	
≥3.89	9.4±1.4	

Continuous variables have been presented as <median and ≥median for purely descriptive reasons; their continuous nature has been appropriately taken into account at the univariate tests; ^T^: Student’s *t*-test; ^A^: Analysis of variance (ANOVA); **§**: Pearson’s correlation coefficient.

Secondary associations that seem worth noting comprise the fact that EPC numbers were significantly correlated with mean arterial pressure (rho=−0.355, p=0.011), hs-CRP (rho=−0.307, p=0.030), HOMA-IR (rho=−0.350, p=0.010), waist circumference (rho=−0.446, p=0.001) and total cholesterol (rho=+0.323, p=0.018).

ADMA was not significantly associated with fasting glucose (Spearman’s rho = +0.070, p=0.619), or HbA1c (Spearman’s rho = −0.002, p=0.988). In contrast ADMA levels were significantly associated with WC (rho=+0.310,p=0.029), median arterial pressure (rho=+0.645,p=0.001), and statin intake (0.67±0.09 μmol/L for subjects taking statins, but 0.78±0.16 μmol/L for those not taking statins, p=0.018 Mann–Whitney-Wilcoxon test for independent samples). A trend towards a positive association between higher ADMA levels and older age was noted (Spearman’s rho=+0.244,p=0.079).

In order to establish which of these correlates were independent predictors of arterial stiffness, multivariate stepwise regression analysis was performed (Table 3). Given the fact that total cholesterol and LDL were collinearly (r=+0.943, p<0.0001) and conceptually interwoven, two alternative models were constructed, beginning with exercise, age, MBP, GFR, ADMA and one of the two collinear variables (LDL-C or total cholesterol). During the stepwise approach, age, fasting plasma glucose, MBP and GFR lost their statistical significance in both cases. As a result, PWV was found to correlate independently and positively with LDL-Cholesterol (coefficient= +0.03, 95%CI: +0.02 to +0.04) and ADMA levels (coefficient= +2.57, 95%CI: +0.84 to +4.31) and negatively with exercise (coefficient= −1.04, 95%CI: -1.53 to −0.55). The same results were replicated in beginning of the analysis with total cholesterol instead of LDL (Table 3).

**Table 3:** Results of the stepwise multivariate linear regression analysis

**Variables**	**Category or Increment**	**Coefficient (95%CI)**	**p**
*Model 1*			
*(Beginning with exercise, age, MBP, LDL, fasting glucose, GFR, ADMA)*			
LDL	1mg/dL increase	+0.03 (+0.02 to +0.04)	<0.001
Exercise	Yes vs. no	−1.04 (−1.53 to −0.55)	<0.001
ADMA	1μmol/L increase	+2.57 (+0.84 to +4.31)	0.004
*Model 2*			
*(Beginning with exercise, age, MBP, total cholesterol, fasting glucose GFR, ADMA)*			
Total cholesterol	1mg/dL increase	+0.03 (+0.02 to +0.04)	<0.001
Exercise	Yes vs. no	−0.91 (−1.41 to −0.40)	0.001
ADMA	1μmol/L increase	+3.80 (+2.08 to +5.53)	<0.001

Variables independently associated with PWV.

## Discussion

In the present study, we investigated the relationship between cardiovascular risk factors and arterial stiffness, with emphasis placed on novel risk factors, as ADMA and EPCs, among subjects with pre-diabetes.

From current knowledge, arterial stiffness represents the combined cumulative effect of traditional cardiovascular risk factors, with the full repertoire of factors implicated to be greatly obscure though. Arterial stiffness is an established predictor of cardiovascular disease, and its increase is associated with a 48% increase of cardiovascular disease risk [26].

According to the results of the present study levels of ADMA, LDL-C, as well as exercise, significantly and directly correlated with PWV in pre-diabetic subjects. The finding of LDL-C correlation with PWV is in accordance with the findings of others [27]. In contrast with the above, other researchers failed to demonstrate any association between aortic stiffness and plasma lipoprotein levels [28]. These conflicting results can be attributed to the different atherosclerotic burden among studied populations, the evaluation of different fractions of lipoproteins, the confounding effect of hypertension, and the use of different techniques/indices for the evaluation of large artery stiffness.

At the molecular level, high LDL-C levels induce endothelial cell dysfunction with subsequent decreased NO bioavailability due to impaired L-arginine transport and metabolism and eNOS uncoupling. Peroxynitrite, the major uncoupling byproduct of eNOs, has been reported to directly damage elastin [11,29,30]. The ensuing decreased NO bioavailability, also increases vascular smooth muscle cell tone, which further aggravates arterial stiffness [11].

Although arterial stiffness could potentially be improved with HMG-coA enzyme inhibition by statins [31] in the present study, a lack of association between statin therapy and PWV was observed. However our findings are in agreement with previously published studies [32].

Several crucial studies have yielded contradictory results in relation to the association of ADMA with a PWV. In our study, ADMA increased arterial stiffness and this is also in agreement with the results of others [18]. Given that NO participates in maintaining the structure of blood vessels [11], it is not surprising that increased expression of a natural nitric oxide synthase (eNOS) inhibitor, such as ADMA, has been linked to vascular stiffening.

In contrast, in the PREVENCION study, ADMA and NMMA (N-MonoMethyl Arginine) were not associated with large artery stiffness [33]. The discrepancies between our analysis and PREVENCION study could be attributed to differences in study design and population characteristics. For example, the sample population in our analysis consisted of individuals with pre-diabetes, carrying a higher atherosclerotic burden. Moreover, in our study we investigated the correlation of cf-PWV with conventional risk factors after multivariate selection of variables including exercise, number of EPC, HOMA index, and use of antihypertensive or hypolipidemic treatment, which were not performed in the PREVENCION study.

In the present study, we did not observe any correlation between EPC count and arterial stiffness. In agreement with our findings, Palombo et al. [27] reported no significant correlation between CD34+/KDR+ counts and c-f PWV in type 1 diabetics. In the same study LDL-C was proved to be independent predictor of c-f PWV in agreement with our findings. Although Yang et al. [34] reported that impaired EPC activity was associated with reduced elasticity among hypertensive subjects, no significant correlation was again demonstrated between the number of circulating EPCs and arterial elasticity indices. It should be underlined though, that our results are not fully comparable with those of the previous study, since subjects with diabetes and smokers were excluded.

In contrast with the above, reduced numbers of circulating EPC have been associated with impaired arterial elasticity among diabetic patients [14,35]. Given that EPC recruitment from bone marrow is NO [13] mediated, it is expected that, as endothelial dysfunction worsens in parallel with glucose levels [36], EPC count would also decrease. Consequently our results are not totally comparable to others, since our study population consists of pre-diabetic individuals.

According to our findings, HbA1c levels did not correlate with PWV at univariate analysis. Fasting glucose levels positively and significantly correlated with PWV at univariate analysis but this correlation was lost at multivariate analysis. A trend for increased PWV across prediabetic categories was observed that did not reach statistical significance though and the above is most possibly due to the small sample size in the present study. It should be noted though that previous studies have shown that hyperglycemia and hyperinsulinemia explain only 30% of arterial changes associated with glucose intolerance [37] and in non-diabetic individuals with lower levels of hyperglycemia no definitive correlation has been proved between arterial stiffness and IGT [38]. From the theoretic point of view it has also been proposed that arterial stiffness in pre-diabetic individuals is, at least partially, NO mediated [38]. Insulin resistance is associated with decreased NO bioavailability while hyperinsulinemia contributes to proliferation of arterial smooth muscle cells [39].

Our results demonstrate that among subjects with pre-diabetes moderate physical activity for at least 150 min per week can reverse arterial stiffness. In agreement with this finding Tanaka et al. [40] have also reported that high levels of physical activity may prevent increases in arterial stiffness in elderly women. In the same study, it was also reported that in addition to aerobic fitness, plasma total cholesterol and plasma LDL-cholesterol levels were significant determinants of central arterial stiffness.

In our sample of prediabetic patients age significantly correlated with increased arterial stiffness at univariate analysis but at multivariate analysis this correlation lost significance. However as previously reported, arterial elasticity is not determined only by the structural components of the vessel wall, but NO can also modify arterial stiffness. Indeed Ngo et al. reported that NO synthesis may represent the basis for increased arterial stiffness in ageing individuals [18]. Even though age is a major determinant of arterial stiffness, several studies reported that increase in large artery stiffness, follows an non linear evolution, implying that an increase in PWV may be observed over the age of 55 years; indeed, in our sample mean age was 50.2 years, well below the 55 year-threshold [41-43].

Although blood pressure is traditionally considered a major determinant of the arterial stiffness, such correlation did not persist in the present study at the multivariate analysis. High levels of systolic blood pressure are believed to increase arterial stiffness due to structural and functional alterations in the arterial media and higher systolic blood pressure levels have been connected with greater increases in arterial stiffness in several prospective studies [44]. Although the relationship of blood pressure with vascular stiffness seems to be bidirectional, investigators of the Framingham Offspring [45] cohort, concluded that vascular stiffness is a precursor rather than the result of hypertension. Other studies have also failed to document SBP as an independent predictor of increased arterial stiffness [46-49] and it has been proposed that increased PWV lays in the basis of increased arterial pressure and not the opposite on a long-term basis. From this point of view the cross sectional nature of our study might have obscured causality between arterial stiffness and MBP that needs time to develop.

A limitation of our study pertains to the relatively small population size. Due to the small sample size, certain secondary observations may not have reached statistical significance; nevertheless, the fact that statistically significant associations emerged and persisted at the multivariate analysis, points to the validity and robustness of the findings presented herein. In addition, the cross-sectional study design does not allow the substantiation of causality, the latter necessitating prospective cohort studies.

Unfortunately in this analysis, qualitative alterations of EPC, such as colony formation, migration or adhesion, were not examined, but in most cases, the number and the functionality of EPC are essentially two sides of the same coin; quantitative and qualitative EPC properties are indeed co-regulated by the same molecular pathways, and consequently decreased EPC count is commonly accompanied with dysfunction [50].

Finally, the use of a control group was not deemed necessary, since the main objective of the present study has been the investigation of correlations between novel and traditional cardiovascular risk factors and arterial stiffness in a specific subpopulation (prediabetics), in which the major, independent determinants of PWV were sought. The validation and replication of previously documented differences (such as comparisons of individuals with pre-diabetes versus controls) was considered redundant and was not performed. Indeed according to the findings of the Hoorn study, severity of arterial stiffness seems to correlate with degree of glucose metabolism impairment with IFG and IGT patients to exhibit increased arterial stiffness that is intermediate between type II frank diabetics and subjects with normal glucose tolerance [5,37] and the above findings have been verified by other studies [6].

Regarding EPC counts, pre-diabetes and diabetes have been directly connected with reduced numbers of circulating EPCs compared with normal glucose tolerance status in several studies [13,14]. Numerous studies have also established that ADMA levels are increased in states of impaired glucose metabolism, in relation to normal subjects, and this increase seems to positively correlate with glycemic category and metabolic control [51,52].

Nevertheless the lack of a control population may be considered a major limitation of the present study. Indeed, an age matched control group may have significantly contributed to the comparison of novel markers (such as EPC and ADMA) between patients with pre-diabetes and healthy subjects. The control group could also have proven that all methods yielded results comparable with the literature. Future studies adopting control groups seem necessary in our setting so to further elaborate and validate the aforementioned findings. In our knowledge, this is the first study to investigate the correlation of traditional and novel cardiovascular risk factors, as AMDA and endothelial progenitor cells and arterial stiffness, as evaluated by PWV, in pre-diabetic individuals.

## Conclusion

In conclusion according to the findings of the present study LDL-cholesterol levels, ADMA levels and physical exercise are strongly associated with PWV among pre-diabetic subjects. In contrast the number of EPCs did not correlate with arterial stiffness. Prospective studies are needed to further elucidate the mechanisms underlying these associations, for determining the impact of lifestyle modifications, or the effects of antilipidemic medications, or insulin sensitizing drugs on PWV [53,54]. Cardiovascular disease is so closely linked to increased PWV, so that identification of the predisposing factors of the latter will improve not only treatment efficacy, focusing on specific destiffening drugs, but also the quality of life of pre-diabetic individuals.

## Abbreviations

EPCs: Endothelial Progenitor Cells; ADMA: Asymmetric Dimethyl-L-Arginine; DDAH: Dimethylarginine Dimethylaminohydrolase; IFG: Impaired Fasting Glucose; IGT: Impaired Glucose Tolerance; FPG: Fasting Plasma Glucose; BMI: Body Mass Index; hsCRP: High Sensitivity C-Reactive Protein; GFR: Glomerular Filtration Rate; HOMA-IR: Homeostasis Model Assessment For Insulin Resistance; LDL: Low Density Lipoprotein; HDL: High Density Lipoprotein; PWV: Pulse Wave Velocity; MAPK: Mitogen-Activated Protein Kinase; PI3K: Phosphoinositide 3 Kinase; eNOS: endothelial NO Synthase; NO: Nitric Oxide; OGTT: Oral Glucose Tolerance Test; FPG: Fasting Plasma Glucose; CAD: Coronary Artery Disease; CVD: Cardiovascular Disease; NYHA: New York Heart Association; DM: Diabetes Mellitus; WHR: Waist-to-Hip Ratio; WHtR: Waist-to-Height Ratio; WC: Waist Circumference; MBP: Mean Blood Pressure; HR: Heart Rate.

## Competing interests

The authors declare that they have no competing interests.

## Authors’ contributions

IP, AM designed the study. IP, AA, AG, SM, KT and GIP performed the study. TS performed the statistical analysis of the study. IP, GD, SF and SR analyzed and interpreted the data. IP and TS wrote the manuscript. All authors read and approved the final manuscript.
